# A One Health perspective on bacterial extracellular vesicles as mediators of antimicrobial resistance spread

**DOI:** 10.1093/ismeco/ycag052

**Published:** 2026-03-11

**Authors:** Haining Huang, Debjyoti Ghosh, Anja Worrich

**Affiliations:** Department of Applied Microbial Ecology, UFZ – Helmholtz Centre for Environmental Research, Permoserstraße 15, 04318 Leipzig, Saxony, Germany; Department of Applied Microbial Ecology, UFZ – Helmholtz Centre for Environmental Research, Permoserstraße 15, 04318 Leipzig, Saxony, Germany; Department of Applied Microbial Ecology, UFZ – Helmholtz Centre for Environmental Research, Permoserstraße 15, 04318 Leipzig, Saxony, Germany; Institute of Biotechnology, Faculty of Environment and Natural Science, Brandenburg University of Technology Cottbus-Senftenberg, Universitätsplatz 1, 01968 Senftenberg, Brandenburg, Germany

**Keywords:** bacterial extracellular vesicles, antimicrobial resistance, horizontal gene transfer, One Health

## Abstract

Antimicrobial resistance (AMR) is a global health threat requiring a One Health approach across human, animal, and environmental sectors. Bacterial extracellular vesicles (BEVs), membrane-bound particles secreted by bacteria, have emerged as potential vectors of antibiotic resistance and mediators of horizontal gene transfer. Found across clinical, agricultural, and natural environments, BEVs carry resistance genes, mobile genetic elements, and virulence factors. They protect genetic cargo, function without direct cell contact, and can cross ecological boundaries more easily than whole bacteria. This review synthesises current knowledge on BEVs in AMR transmission, highlights their cross-sector potential, and identifies key research gaps. Recognising their role is essential for improving AMR surveillance and informing future mitigation strategies.

## BEVs: from formation to function

Extracellular vesicles (EVs) are nanoscale particles typically ranging from 30 to 200 nm [1–3] that are released by organisms across all domains of life, including prokaryotes, eukaryotes, and archaea [4]. In bacteria, these vesicles are commonly referred to as bacterial extracellular vesicles (BEVs), which have received growing attention for their roles in various interactions. These include cell-to-cell communication [5], bacterial antagonistic interactions mediated by the delivery of antimicrobial factors such as bacteriocins and lytic enzymes [6–8], pathogenesis [9–11], and horizontal gene transfer (HGT) [12, 13]. In gram-negative bacteria, BEVs are primarily generated through two mechanisms: blebbing of the outer membrane or explosive cell lysis, in which fragmented membrane components reassemble into vesicles [2, 3, 14, 15]. Explosive lysis is typically initiated by genotoxic stress that activates prophage-encoded endolysins, leading to peptidoglycan degradation and subsequent membrane rupture [15, 16]. In gram-positive bacteria, BEVs are predominantly produced via the enzymatic action of endolysins or autolysins, which cause localised weakening of the peptidoglycan layer, causing the protrusion of the cytoplasmic membrane and the subsequent release of vesicles, a process known as “bubbling cell death” [3, 17].

**Figure 1:**
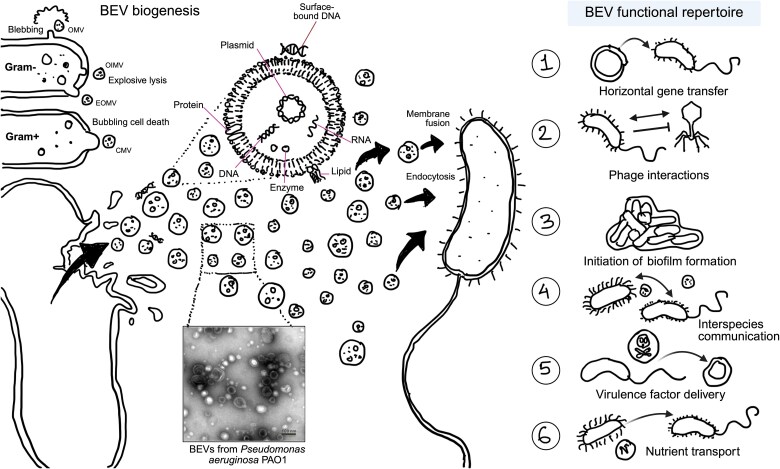
Biogenesis, structural characteristics and functional repertoire of BEVs. The figure illustrates the proposed mechanisms of BEV formation, the typical vesicle structure with diverse cargo molecules (including proteins, nucleic acids, and metabolites), and the broad functional roles of BEVs.

Due to the distinct biogenesis pathways, BEVs encapsulate a diverse array of bioactive molecules, collectively referred to as “cargo.” This cargo includes nucleic acids, proteins, lipids, and metabolites (Fig. 1). This makes them mediators of a broad range of functions [2, 3], including intercellular communication [18, 19], nutrient and electron transfer [20, 21], phage interactions [7, 22, 23] and biofilm formation [24]. Additionally, BEVs play a potential role in bacterial pathogenicity by selectively incorporating virulence-associated molecules such as toxins and effectors and delivering them to nearby or remote host cells, enabling pathogens to interact with host cells without direct cell contact [9, 14].

This vesicle-mediated strategy enhances the virulence potential of pathogens and is involved in the pathogenesis of various infectious diseases affecting both humans and animals. For example, a toxin released via BEVs from *Campylobacter jejuni* induces cytotoxic and distending effects in tissue culture cells [10]. BEVs secreted by the opportunistic human pathogen *Pseudomonas aeruginosa* deliver multiple virulence factors such as alkaline phosphatase, haemolytic phospholipase C, and CFTR inhibitory factor directly into the host cell cytoplasm through fusion of BEVs with lipid rafts within the host plasma membrane [9]. Opportunistic human pathogens like *Streptococcus pneumoniae* release BEVs containing virulence factors such as the protein kinase *StkP* into host cells, which ultimately impairs epithelial barrier function [11].

In addition to their role in bacterial pathogenicity, BEVs also contribute to the spread of AMR as they transport genetic material containing antibiotic resistance genes (ARGs). The following sections focus on ARGs carried by BEVs across human, animal, and environmental sectors, emphasising their relevance within an AMR One Health context.

## BEVs as mediators of AMR in humans, animals and the environment

Antimicrobial resistance (AMR) is a critical global health threat. In 2021, AMR-related infections caused 4.71 million deaths, of which 1.14 million were directly attributed to bacterial AMR [25]. AMR is a One Health issue as it is prevalent in humans, animals, and environmental sectors and may cross between these sectors [26]. Antibiotic resistant bacteria and their ARGs can spread among livestock, wildlife, humans, and environmental reservoirs such as soil and water, potentially creating complex transmission networks that transcend traditional sectoral boundaries [26]. Understanding the mechanisms facilitating this cross-sectoral ARGs transmission is essential for developing effective One Health strategies to combat AMR.

**Figure 2:**
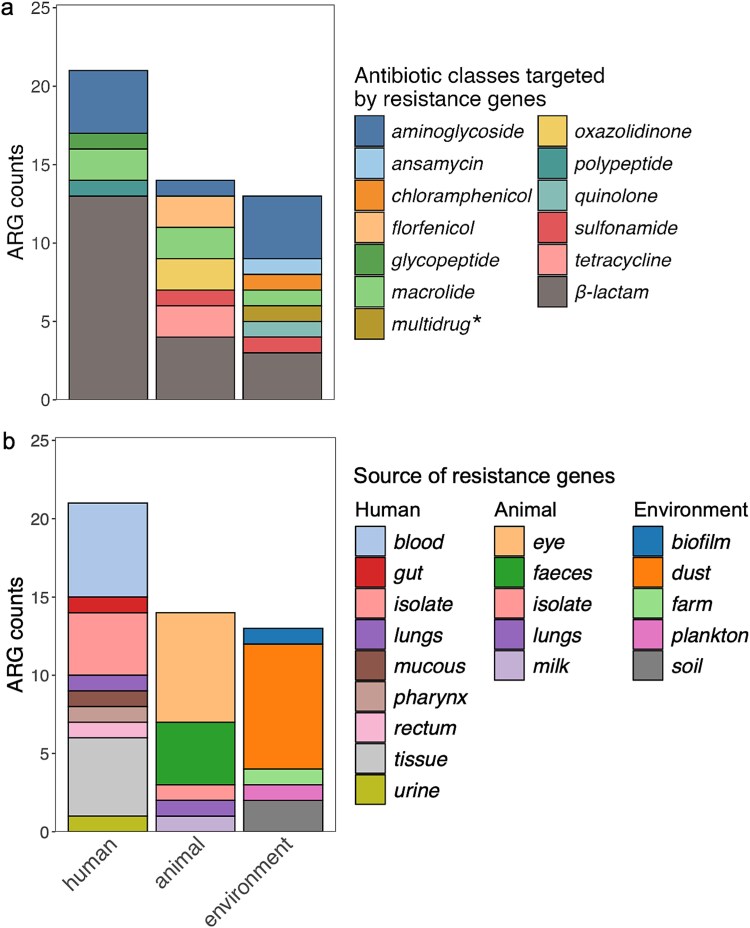
Distribution of ARGs transmitted via BEVs across human, animal, and environmental sectors based on published literature. (a) Counts of reported ARGs grouped by antibiotic class across human, animal, and environmental sectors (*multidrug ARGs confer resistance to multiple antibiotic classes); (b) Counts of reported ARGs according to the source of sampled specimens within each sector.

Current evidence suggests that BEVs may contribute to AMR through two distinct mechanisms: HGT of ARGs and direct protection. First, with respect to HGT, BEVs can carry diverse ARGs, (Fig. 2, Supplementary Table S1) and facilitate their transfer between bacteria, thereby allowing the permanent acquisition of resistance traits by recipient bacteria. Second, beyond genetic transfer, BEVs can provide immediate but transient protection towards antibiotics by delivering antibiotic-degrading enzymes [27] or sequestering antimicrobial peptides [28]. Furthermore, BEVs can function as decoys for antibiotics through a passive mechanism often mediated by surface proteins, binding and encapsulating the drugs and thereby reducing the effective antibiotic concentration reaching the bacterial surface [29]. These properties highlight the potential of BEVs to act as multifaceted agents in the spread and mediation of AMR.

## The human sector

BEVs have been detected in various human-associated environments. In human faeces they have been shown to reach concentrations between ~10^10^ and 10^11^ particles per gram (Table 1). Based on a metagenomics survey on BEVs extracted from human faeces, BEV-associated DNA was derived from 39 bacterial phyla, with *Bacillota, Pseudomonadota, Bacteroidota, Verrucomicrobiota*, and *Actinomycetota* being the dominant sources [30]. A total of 122 ARG subtypes belonging to 16 antibiotic classes were identified in faecal-derived BEVs, and ARGs conferring resistance to tetracyclines, macrolide–lincosamide–streptogramin and aminoglycosides accounted for 77.62% of the total [30]. This finding highlights a vast diversity of ARGs carried by BEVs. BEVs secreted from *Haemophilus influenzae* and *Moraxella catarrhalis* strains carrying the β-lactam-resistance gene *bla*_TEM-1_ protected *Streptococcus pyogenes* against the effects of amoxicillin [31]. BEVs secreted by multidrug-resistant *Enterococcus faecium* and *Salmonella enterica* harbour ARGs facilitating resistance to vancomycin- (*vanA*) and polymyxin antibiotics [32, 33]. Although many studies have been conducted on ARGs transferred by BEVs, the majority of them focus on genes conferring resistance against β-lactams, and hence, other ARGs in BEVs remain understudied.

**Table 1:** Selected representative studies reporting the characteristics of EVs (size, abundance and cargo) across diverse human, animal, and environmental sectors.

Environment	Sample source	Size information	Abundance	Cargo	Reference
Host-associated	Human faeces	~40–160 nm	~10^10^–10^11^ /g	DNA, ARGs	[30]
	Animal faeces	~20–500 nm	~10^12^ /g	DNA, ARGs	[51]
Terrestrial	Dust (indoor & agricultural)	~50–200 nm	~10^7^–10^11^ /g	DNA，ARGs, Allergens	[54–58]
	Soil	~20–500 nm	~10^7^–10^9^ /g	DNA, ARGs, Protein	[30, 51, 84]
Aquatic	Wastewater	~20–500 nm	~10^4^–10^10^ /ml	DNA, ARGs	[30, 51, 60]
	Ocean	~30–250 nm	~10^4^–10^6^ /ml	DNA	[23, 63, 64]
	Freshwater	~40–160 nm	~10^5^–10^6^ /ml	DNA, Signaling molecules	[85]

Note: This table focuses on in situ studies involving the direct isolation and quantification of BEVs from raw environmental and biological samples, excluding data derived solely from laboratory-cultured isolates.

BEVs have the ability to transmit ARGs via HGT [34, 35]. They have been reported to transport a diverse array of plasmids encoding resistance genes to β-lactam [36–38], carbapenem [12, 13, 39–43], spectinomycin [44], kanamycin [44], gentamicin [45] and vancomycin [46]. BEVs secreted by clinical isolates of *Acinetobacter baumannii* showed intraspecific transfer of a carbapenemase gene *(bla*_OXA-24_), resulting in resistance to β-lactam antibiotics in the recipient strains [40]. Some of these resistance genes, e.g. *bla*_CTX-M-55_, *bla*_KPC-2_ and *floR,* are classified as high-risk ARGs by the WHO [47, 48]. Future studies are necessary to determine how BEV-mediated transmission of ARGs in human-associated microbiomes, especially the gut, contributes to the emergence of multidrug-resistant pathogens and compromises the efficacy of therapeutic approaches using antibiotics.

## The animal sector

The use of antibiotics in livestock has resulted in the development and spread of AMR within animal-associated bacterial populations [49]. BEVs derived from animal sources are predominantly derived from chicken [28, 50] and swine [51, 52], with some carrying high-risk ARGs [47, 48]. However, relatively few studies have investigated BEVs produced in animal-derived bacteria compared to those produced by human-derived bacteria, primarily due to the increasing focus on the role of BEVs in human health and medicine.

BEVs secreted by cultivated *Staphylococcus aureus* isolated from chicken meat contain the β-lactam resistance gene *blaZ* when exposed to antibiotics [50], enabling the bacteria to protect themselves and other commensal bacteria [28, 50]. Similarly, BEVs secreted by bacterial strains isolated from chicken, especially when cultured in the presence of ampicillin, demonstrated enhanced potency in degrading β-lactam antibiotics as well as protection from imipenem, cefotaxime, and methicillin [53]. A metagenomic analysis of BEVs extracted from swine faeces revealed that BEV-associated DNA was derived from 18 phyla, with *Pseudomonadota, Bacteroidota, Bacillota*, and *Actinomycetota* among the predominant groups. These BEVs were found to carry diverse resistance genes, including phenicol-oxazolidinone (*optrA, poxtA*), multidrug (*adeF, TolC*), and tetracycline-resistance genes (*tetO, tetT, tetD*) [51]. Furthermore, BEVs secreted by *Enterococcus faecalis* isolated from swine faeces mediated the transfer of the phenicol-oxazolidinone resistance gene *optrA* and plasmid-borne ARGs to human-derived *Enterococcus* strains. This demonstrates the occurrence of BEV-mediated gene exchange among Gram-positive bacteria and suggests a route for the spread of ARGs among potential zoonotic pathogens [52]. The MICs for three different antibiotics increased by a factor of 8 after exposure of human *Enterococcus* strains to BEVs secreted by pig-isolated *E. faecalis*, strongly suggesting the occurrence of BEV-mediated HGT. This vesicle-mediated HGT between animal- and human-derived bacterial isolates suggests that BEVs may be involved in cross-sectoral transfer of genetic material [52].

## The environmental sector

BEVs have been detected in a wide range of environmental matrices, such as dust [54–59], soil [30, 51], wastewater [30, 51, 60], freshwater [61, 62], and coastal and open-ocean seawater [23, 63–65]. These observations collectively indicate that BEVs are ubiquitous across various natural ecosystems. Although reports on BEVs in diverse ecosystems are increasing steadily, their functional roles remain largely unexplored. This is primarily due to the complexity of environmental systems, which are characterised by high microbial diversity, fluctuating physicochemical conditions (e.g. temperature, pH, and nutrient availability), and intricate biotic and abiotic interactions. Technically, it is challenging to isolate pure BEVs because they overlap in size and density with non-vesicular particles such as viruses and inorganic colloids, leading to their co-extraction [23, 66, 67]. Therefore, adhering to consensus guidelines (e.g. MISEV) is critical to distinguish BEVs from environmental background noise and ensure data reproducibility [68]. In complex environmental matrices, the limited availability of appropriate experimental tools and model systems currently hinders progress in understanding BEV functions in environmental contexts.

In seawater, the concentration of EVs reaches ~10^4^–10^6^ particles per millilitre (Table 1). Metagenomics performed on EV-associated DNA in both coastal and open ocean waters revealed that EVs are potentially produced by microorganisms spanning all three domains of life, including representatives from at least 33 phyla [23], with the majority of DNA-containing EVs being produced by prokaryotes [63]. Copiotrophic and oligotrophic bacteria, such as *Flavobacterium* and *Pelagibacter,* were shown to be the dominant sources of BEV-associated DNA in seawater [63].

Several studies on BEVs focused on indoor dust, due to its strong association with human health. Dust-associated BEVs from restaurants, kindergartens, dormitories, and vehicles showed abundances ranging from ~10^7^ to 10^11^ particles per gram of dust (Table 1). A total of 241 ARG subtypes conferring resistance to 16 classes of antibiotics were found in dust-associated BEVs [59]. Among them, multidrug resistance genes were the most dominant ARGs detected. Compared to the bacterial communities present in dust, BEVs exhibited a significantly higher relative abundance of these ARGs, suggesting selective enrichment. For instance, multidrug efflux pump genes such as *mexK* and *mexW* were markedly more abundant in the vesicle fraction compared to the bacterial fraction. This suggests that BEVs may selectively package and transport specific ARGs [59]. However, such findings warrant cautious interpretation. As highlighted in metagenomic surveys, the detection of DNA sequences, especially highly abundant housekeeping genes like *EF-Tu* or *rpoB*, does not imply selective packaging or functional resistance. These sequences may be encapsulated due to stochastic packaging of chromosomal fragments. Furthermore, unlike dedicated resistance genes (e.g. efflux pumps), resistance in housekeeping genes relies on specific point mutations rather than mere gene presence. Thus, differentiating between selectively enriched cargo and random chromosomal background remains a critical challenge for future studies.

Urban wastewater treatment plants are critical monitoring points for AMR and often described as the “gut” of the city. EVs are present in wastewater at concentrations ranging from ~10^4^ to 10^10^ particles per millilitre. Metagenomic analysis identified *Pseudomonadota, Bacteroidota, Bacillota* and *Actinomycetota* as the dominant sources of BEV-associated DNA [30]. BEVs in wastewater influent were primarily derived from prokaryotes and carried 128 ARGs belonging to 11 antibiotic classes [30]. Among these, the most prevalent ones were tetracycline, macrolide-lincosamide-streptogramin, multidrug, and β-lactam resistance genes [30]. Antibiotic exposure alters the composition of BEV-associated ARGs, supporting the notion that BEVs may actively contribute to mediating AMR in bacterial communities [60]. Soil has also been identified as a rich reservoir of BEVs, with concentrations ranging from ~10^7^ to 10^9^ particles per gram (Table 1). In BEVs isolated from public park soils, a total of 17 ARGs were identified [30], while 16 ARGs were detected in BEVs collected from swine farm soils that were mainly associated with fluoroquinolone, tetracycline, and macrolide resistance [51]. These genes are commonly detected in swine faeces, supporting the idea of animal manure serving as a major transmission pathway for ARGs from animals to the environment via the application of manure as fertiliser on arable land [49].

## BEV-mediated ARGs transfer

BEV populations can collectively encapsulate DNA representing nearly the entire bacterial chromosome, with more than 99% of the detected genetic material in BEVs originating from chromosomal DNA [69]. However, individual BEVs contain only DNA segments rather than intact genomes. The extent to which this cargo results from active packaging processes versus random presence remains a topic of debate. Although chromosomal DNA is predominant, cargo incorporation appears to be largely determined by cytoplasmic abundance. For instance, the efficiency of plasmid packaging is positively correlated with plasmid copy number resulting in the accumulation of high-copy number plasmids [37, 70]. Thus, the composition of BEV cargo likely resembles the cytoplasmic composition of the producer cells and the availability of DNA fragments occurring prior to vesicle formation. Consistent with this model, exposure to antibiotics such as enrofloxacin induces SOS responses that promote the accumulation of intracellular DNA fragments via antibiotic-mediated DNA damage. The enhanced availability of these fragments facilitates their encapsulation during vesicle formation, resulting in a significantly higher DNA load within vesicles [71].

The potential of BEVs to serve as vectors for ARG dissemination is largely attributed to their unique biological properties. Structurally, the lipid bilayer of BEVs serves as a protective shield, preserving the integrity of encapsulated DNA against extracellular nucleases [13, 53]. This protection theoretically enables genetic material to persist in harsh environments for extended periods, although comprehensive data on the long-term stability of BEV-associated DNA across diverse environmental matrices remains limited. Additionally, the nano-scale size of BEVs facilitates efficient diffusion, enabling them to move through liquid pathways, such as pore water in soil. This mobility allows for genetic exchange without the need for direct cell-to-cell contact, thereby overcoming the spatial isolation of bacterial populations often found in complex matrices [19].

**Figure 3:**
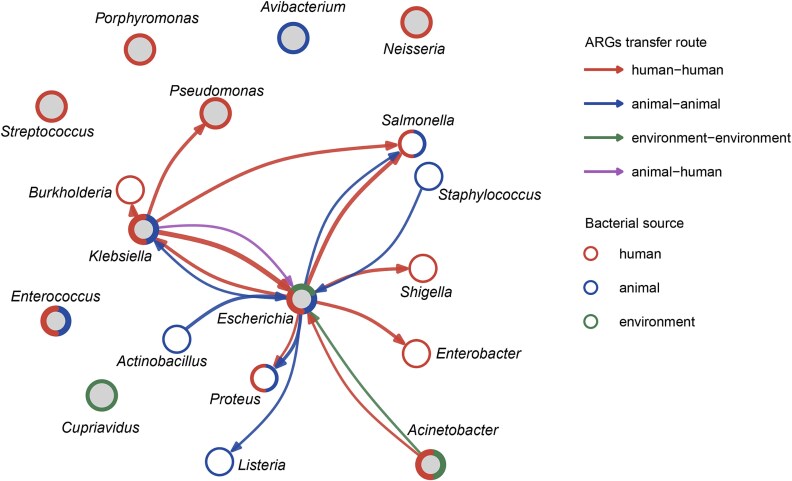
Comprehensive network representation of bacterial genera involved in BEV-mediated horizontal gene transfer (HGT). The network is constructed based on a systematic review of 31 published studies (identifying 106 transfer events) demonstrating BEV-mediated HGT among bacterial strains isolated from humans, animals or the environment. Nodes are shown as solid circles representing different bacterial genera. Edge colours indicate the origin of the donor and recipient isolates: red lines represent transfers between isolates of human origin; blue lines represent transfers between isolates of animal origin; green lines represent transfers between environmental isolates; and purple lines represent cross-sector transfers between animal- and human-derived isolates. The outer border colour around the circle indicates the sector from which isolates were obtained sector, and bold borders around circles filled with grey denote genera where intra-genus transfers were observed. Cross-sector linkages highlight the potential for BEVs to contribute to the spread of AMR across One Health sectors.

BEVs have been shown to carry plasmids ranging from 2.1 kb to 162.9 kb, harbouring various ARGs and virulence factors [42, 72, 73]. Self-replicating plasmids represent highly efficient vehicles for gene transfer, as they can be maintained autonomously in recipient cells, whereas chromosomal DNA is typically packaged as fragments that require recombination for stable integration [69]. In contrast, proteins carried by BEVs can only provide transient protection, as they are not genetically inherited [28, 50]. BEVs have been shown to mediate plasmid transfer both within a genus and across different genera (Fig. 3), highlighting their potential role in facilitating gene exchange across taxonomic boundaries. They mediate HGT among a broad spectrum of bacterial genera, including Gram-negative genera such as *Escherichia, Pseudomonas*, and *Salmonella*, as well as Gram-positive genera such as *Staphylococcus, Enterococcus*, and *Streptococcus* (Fig. 3). Among those genera, *Escherichia* is the most studied organism, acting both as donor and recipient across diverse taxa. *Escherichia* BEVs not only facilitate plasmid transfer within the genus but also enable cross-genus transfer to *Shigella, Enterobacter, Salmonella, Pseudomonas, Proteus*, and *Klebsiella*. Such observations of intra- and inter-genus HGT indicate a conserved mechanism of genetic exchange. Moreover, BEVs can cross phylogenetic barriers between Gram-negative and Gram-positive bacteria despite the huge differences in their membrane compositions. For example, plasmid exchange between Gram-positive genera (*Staphylococcus, Listeria*) and Gram-negative *Escherichia* via BEVs [53, 74] highlights their ability to disseminate mobile genetic elements across broad evolutionary distances.

Despite clear evidence of transfer, the specific mechanisms governing recipient uptake and host specificity remain a major knowledge gap. While processes such as membrane fusion and endocytosis have been proposed [2, 19], the factors that define efficient recipients remain poorly understood. In terms of efficiency, the frequency of BEV-mediated HGT, here defined as the ratio of plasmid-carrying bacteria to the total number of recipient cells, varies significantly with environmental conditions. Reported frequencies range from ~10^−9^ under antibiotic-free culture conditions [36] to as high as 10^−4^ ~ 10^−3^ under antibiotic pressure or in simulated intestinal environments [71, 75]. Our comparative analysis (Supplementary Table S2) reveals a clear hierarchy of transfer efficiency. In studies directly comparing BEV-mediated transfer with conjugation and transformation, BEV-mediated transfer is generally less efficient than conjugation but exceeds natural transformation by free DNA. Remarkably, in multiple studies, BEV-mediated transfer occurred successfully with frequencies ranging from 10^−8^ ~ 10^−4^, while free plasmid transformation controls yielded no detectable transformants (Supplementary Table S2). This suggests that BEV-mediated HGT may be a more important driver of genetic exchange in environments where conjugation is limited by conditions that restrict direct cell-to-cell contact. However, these comparisons should be interpreted with caution, as absolute transfer frequencies are not directly comparable across studies due to differences in experimental design (e.g. incubation times and donor-to-recipient ratios).

The transfer of ARGs by BEVs is clinically relevant due to its potential to accelerate the spread of resistance among pathogenic bacteria. Recent studies showed that BEVs secreted by the zoonotic pathogen *E. coli* O157:H7 can transfer the plasmid pUC18, encoding an ampicillin resistance-conferring β-lactamase gene, to *S. enterica* serovar Enteritidis (ATCC 13076) and *E. coli* JM109 [12]. Carbapenem-resistant *K. pneumoniae* strains secrete BEVs carrying the *bla*-NDM-1 gene, facilitating the transfer of this high-risk resistance determinant to other *K. pneumoniae* strains, including hypervirulent lineages [42]. These transfers not only facilitate the dissemination of resistance within species but also contribute to the convergence of AMR and virulence, which greatly complicates clinical treatments and infection control. Multiple bacterial species capable of BEV-mediated HGT have been identified among isolates from human and animal sources, showing that this vesicle-mediated gene transfer mechanism is not restricted to a single reservoir and could therefore contribute to the exchange of AMR determinants at the human–animal interface. However, it is important to note that much of the current knowledge is based on studies using *E. coli* as a donor or recipient model. This literature bias may skew our understanding of HGT dynamics in other bacterial species. Consequently, the specific role of BEVs derived from diverse non-model taxa in AMR transmission remains largely unexplored.

## One Health implications

The One Health approach highlights the interconnectedness of human, animal, and environmental health, providing a framework for understanding the routes of AMR transmission across sectors. Produced by virtually all bacterial species in all One Health sectors, BEVs have been shown to carry ARGs, mobile genetic elements, and virulence factors, making them highly relevant as a potential dissemination route for AMR across these sectors [76]. Currently, standardized methods capable of tracking and confirming intersectoral transfers are missing. While direct empirical evidence of BEV dissemination across sectors remains limited, their physical properties likely foster enhanced mobility compared to whole cells. Analogous to viruses and nanoplastics [77–79], the nanometric size of BEVs minimizes the influence of gravitational settling and allows for transport through narrow pore spaces in complex matrices that typically act as filtration barriers for larger bacterial cells [77–79]. Consequently, BEVs are theoretically better able to overcome physical dispersal barriers. Moreover, their robust lipid bilayer protects the encapsulated cargo from environmental stressors and enzymatic degradation, thereby enhancing their environmental persistence and maintaining their functional integrity. The risk of cross-sectoral spread via BEV-mediated horizontal gene transfer is further exacerbated by several aspects: unlike conjugation, it does not require direct cell-to-cell contact; unlike natural transformation, it bypasses the reliance on free DNA, which is often degraded in the environment; and unlike phage-mediated transduction, which is generally constrained by host specificity, BEVs can interact with a wider range of recipient cells, facilitating gene transfer across diverse taxa, as demonstrated by evidence of inter-genus transfers [80].

**Figure 4:**
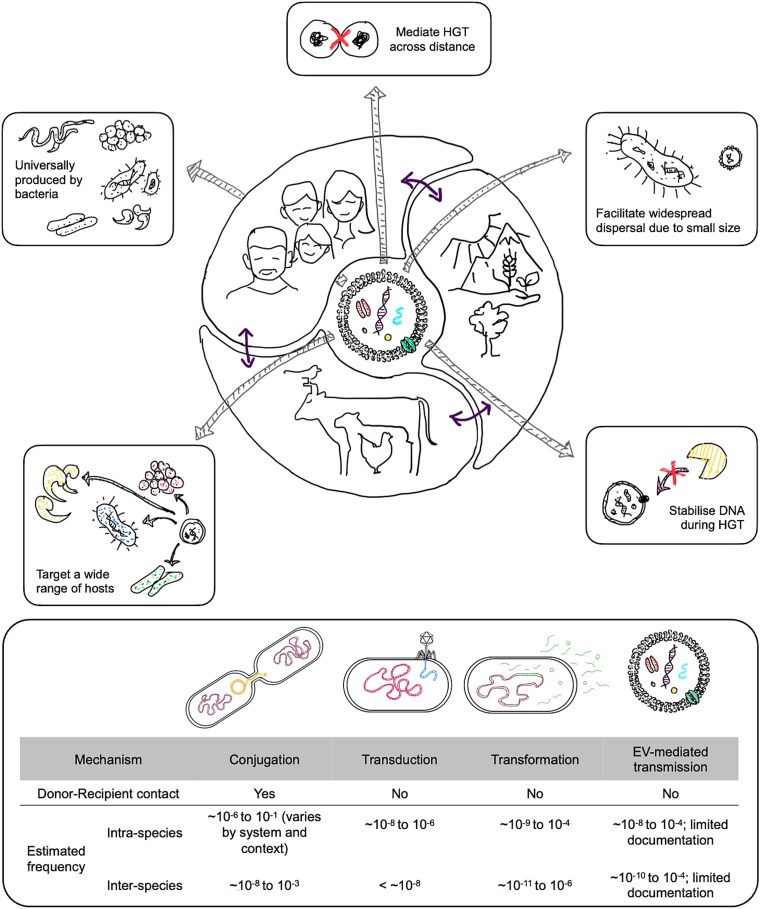
Advantages of BEV-mediated horizontal gene transfers across different sectors. BEVs can mediate cross-sectoral gene transfer independently of direct cell-to-cell contact, extracellular DNA stability, or specific recipient cells as compared to other HGT mechanisms (conjugation, transduction and transformation).

The advantages of BEV-mediated genetic transfer have been demonstrated in studies in the marine environment, where direct cell-to-cell contact is comparatively rare and free-living bacteria lack the genetic basis to initiate natural transformation [81]. Lücking et al. hypothesised that BEVs are major drivers of genetic exchange in the ocean, based on a metagenomics datasets derived from size-fractionated and DNase-treated samples, in which 39% of reads were classified as non-viral, despite the ocean being an environment where viruses are considered the most abundant DNA-containing biological entities [82]. From a One Health perspective, this raises questions about the role of BEVs in mediating the dissemination of resistance determinants across ecological and epidemiological boundaries separating environmental, animal, and human reservoirs (Fig. 4).

## Future perspectives

Produced by a wide range of bacterial species, BEVs are stable carriers of genetic material involved in HGT, enabling transfer without the need for direct cell-to-cell contact, uptake of free DNA, or specific host compatibility. That being the case, BEVs may potentially facilitate gene flow across ecological boundaries, linking distinct environments and microbial communities. Understanding their role in the dissemination of AMR is therefore crucial from a One Health perspective, as it sheds light on how resistance genes may move between humans, animals, and the environment, where such exchanges can have huge implications for public health, animal health, and ecosystem integrity. However, important knowledge gaps remain and must be addressed to fully assess their role in the spread of AMR across sectors. A considerable variability in their cargo composition has been reported due to the differences in their biogenesis [3]. Even though there are reports about plasmid-mediated gene transfer via BEVs, the proportion of vesicles that carry plasmid DNA under natural or experimental conditions remains unclear. Determining this proportion is essential for assessing the extent to which BEVs contribute to the spread of AMR and for understanding whether the “packaging” takes place randomly or can be triggered by external stimuli. In addition, BEV-mediated gene transfer is not uniformly effective across bacterial taxa. The possibility of recipient specificity causing this non-uniform genetic transmission has been suggested, but the underlying mechanisms remain poorly characterised [52, 83]. Further research is needed to determine the host range and compatibility of BEVs, including the potential role of membrane receptor recognition, surface charge interactions, and vesicle uptake pathways such as membrane fusion, endocytosis, or pili-mediated contact. This includes not only transfer between bacterial species but also the potential delivery of genetic material or other cargo to eukaryotic host cells. While direct evidence of such inter-domain transfers is limited, the possibility warrants further investigation.

Studies are often conducted on BEVs from bacterial isolates, yet their role and impact within complex microbial communities, especially in the context of AMR dissemination, remain poorly understood. To fully grasp their contribution to AMR spread, it is essential to investigate BEV dynamics, interactions, and stability in natural environments and multispecies communities.

## Supplementary Material

ycag052_Supplementary_material_Huang

## Data Availability

Data sharing is not applicable to this article as no datasets were generated or analysed during the current study.
