# Supplementary material for: A One Health perspective on bacterial extracellular vesicles as mediators of antimicrobial resistance spread
Source: ISME Commun. 2026 Mar 11;6(1):ycag052. doi: 10.1093/ismeco/ycag052 (PMC13064641; doi:10.1093/ismeco/ycag052)
Supplement: ycag052_Supplementary_material_Huang [file ycag052_supplementary_material_huang.docx]

Supplementary Material

A One Health Perspective on Bacterial Extracellular Vesicles as Mediators of Antimicrobial Resistance Spread

Haining Huang^1^, Debjyoti Ghosh^1^, Anja Worrich^1,2^*

^1^ Department of Applied Microbial Ecology, UFZ - Helmholtz Centre for Environmental Research, Permoserstraße 15, 04318 Leipzig, Saxony, Germany

^2^ Institute of Biotechnology, Faculty of Environment and Natural Science, Brandenburg University of Technology Cottbus-Senftenberg, Universitätsplatz 1, 01968 Senftenberg, Brandenburg, Germany

Corresponding author. Brandenburg University of Technology Cottbus-Senftenberg, Faculty of Environment and Natural Science, Chair of Microbiology, Universitätsplatz 1, 01968 Senftenberg, Brandenburg, Germany. Email: worrich@b-tu.de

# Extended data

Data source

A systematic literature search was conducted in June 2024 to identify research articles specifically addressing bacterial extracellular vesicle (BEV)-mediated horizontal gene transfer (HGT). Two major databases, PubMed and Web of Science, were searched using a comprehensive combination of keywords based on Boolean logic: (Topic A) AND (Topic B). Topic A included terms related to vesicles: 'outer membrane vesicles', 'membrane vesicles', 'extracellular vesicles', or 'bacterial membrane vesicles'. Topic B included terms related to genetic exchange: 'horizontal gene transfer', 'gene transfer', 'gene exchange', or 'antibiotic resistant'.

The initial search yielded a total of 638 records (371 from PubMed and 267 from Web of Science). After removing duplicates, 312 unique records were screened based on titles and abstracts. Studies were excluded if they: (1) focused solely on EV characterization without evidence of gene transfer; (2) investigated non-bacterial EVs; or (3) were review articles.

Finally, 31 research articles met all inclusion criteria and were selected for detailed analysis. From these studies, 106 valid data entries representing BEV-mediated HGT events from donor to recipient bacteria were extracted. For each HGT event, we systematically documented comprehensive information including: (i) the relevant source (human, animal, or environment); (ii) BEVs isolation and purification methods; (iii) particle detection methods and size characteristics; (iv) donor and recipient bacterial genera and species with their Gram-staining properties; (v) the relationship between donor and recipient (inter-species vs. intra-species); (vi) type and characteristics of transferred genetic elements (plasmid vs. chromosomal DNA, plasmid size, marker genes); (vii) antibiotic resistance genes; (viii) experimental methods for tracking EV-bacteria interactions and detecting gene transfer (PCR, phenotypic analysis, or genomic sequencing); and (ix) HGT frequencies when reported.

This comprehensive and systematic dataset forms the basis of our analysis and distinguishes this review by providing detailed insights into the diversity of species involved in BEV-mediated HGT from the various One Health sectors.

While the recent comprehensive review by De Langhe et al. [1] provided a broad bibliometric mapping of the general bacterial EV research landscape, our work adopts a distinct and specialized focus by specifically prioritizing BEV-mediated HGT events, extracting transfer-related information and putting it into a One Health perspective for AMR.


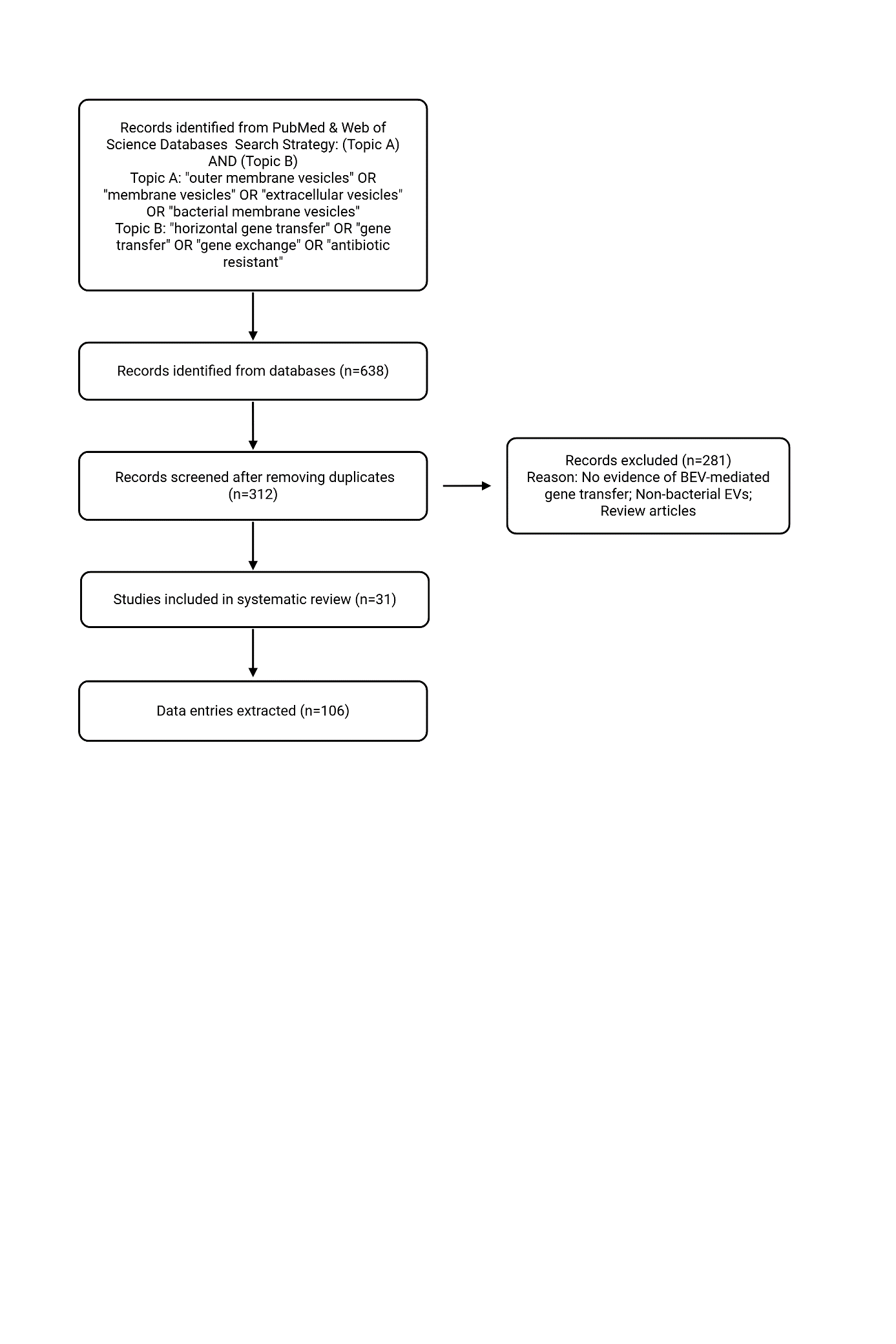


Figure S1: PRISMA flow diagram of the literature selection process. A systematic search was conducted in PubMed and Web of Science databases (June 2024) using keywords related to bacterial extracellular vesicles (Topic A) and horizontal gene transfer (Topic B). The initial search identified 638 records. After removing duplicates and applying exclusion criteria (studies solely on EV characterization, non-bacterial EVs, or lacking evidence of gene transfer), 31 studies were selected for detailed analysis, yielding 106 valid data entries.

# Supplementary Tables

Table S1: Overview of studies on ARGs reported in BEVs across human, animal and environmental sectors

| Year | Title | Source | Source_detailed | ARGs type |  |
| --- | --- | --- | --- | --- | --- |
| 1989 | Export and intercellular transfer of DNA via membrane blebs of *Neisseria gonorrhoeae* | human | human - gut | β-lactam |  |
| 2004 | DNA-containing membrane vesicles of *Pseudomonas aeruginosa* PAO1 and their genetic transformation potential | human | human - tissue | β-lactam |  |
| 2011 | Horizontal transfer of the OXA-24 carbapenemase gene via outer membrane vesicles: a new mechanism of dissemination of carbapenem resistance genes in *Acinetobacter baumannii* | human | human - blood | β-lactam |  |
| 2014 | Gene transfer potential of outer membrane vesicles of *Acinetobacter baylyi* and effects of stress on vesiculation | environment | soil | β-lactam |  |
| 2015 | Functional Advantages of *Porphyromonas gingivalis* Vesicles | human | human - tissue | macrolide |  |
| 2017 | *Acinetobacter baumannii* transfers the blaNDM-1 gene via outer membrane vesicles | human | human - blood | β-lactam, aminoglycoside |  |
|  |  |  |  |  |  |
| 2020 | Dissemination of the blaCTX-M-15 gene among *Enterobacteriaceae* via outer membrane vesicles | human | human - blood | β-lactam |  |
|  |  |  | human - urine | β-lactam |  |
| 2021 | Outer Membrane Vesicles Derived from *Klebsiella pneumoniae* Are a Driving Force for Horizontal Gene Transfer | human | human - tissue | β-lactam |  |
| 2021 | On the Offensive: the Role of Outer Membrane Vesicles in the Successful Dissemination of New Delhi Metallo-β-lactamase (NDM-1) | human | human - isolate | β-lactam |  |
| 2021 | “One for All”: Functional Transfer of OMV-Mediated Polymyxin B Resistance From *Salmonella enterica* sv. Typhi ΔtolR and ΔdegS to Susceptible Bacteria | human | human - isolate | polypeptide |  |
| 2022 | Outer membrane vesicles-transmitted virulence genes mediate the emergence of new antimicrobial-resistant hypervirulent *Klebsiella pneumoniae* | human | human - pharynx | β-lactam |  |
| 2022 | Outer Membrane Vesicles of Avian Pathogenic Escherichia coli Mediate the Horizontal Transmission of blaCTX-M-55 | animal | bird - isolate | β-lactam |  |
| 2022 | Comparative Genomics Analysis and Outer Membrane Vesicle-Mediated Horizontal Antibiotic-Resistance Gene Transfer in *Avibacterium paragallinarum* | animal | bird - eye | aminoglycoside, β-lactam, macrolide, oxazolidinone, sulfonamide, tetracycline |  |
|  |  |  |  |  |  |
|  |  |  |  |  |  |
|  |  |  |  |  |  |
|  |  |  |  |  |  |
| 2022 | Membrane vesicles from antibiotic‐resistant *Staphylococcus aureus* transfer antibiotic‐resistance to antibiotic‐susceptible *Escherichia coli* | animal | bird - eye | β-lactam |  |
| 2022 | T6SS secretes an LPS-binding effector to recruit OMVs for exploitative competition and horizontal gene transfer | environment | soil | aminoglycoside |  |
| 2022 | Horizontal gene transfer via OMVs co-carrying virulence and antimicrobial-resistant genes is a novel way for the dissemination of carbapenem-resistant hypervirulent *Klebsiella pneumoniae* | human | human - tissue | β-lactam, aminoglycoside |  |
|  |  |  |  |  |  |
| 2022 | Dissemination of virulence and resistance genes among *Klebsiella pneumoniae* via outer membrane vesicle: An important plasmid transfer mechanism to promote the emergence of carbapenem-resistant hypervirulent *Klebsiella pneumoniae* | animal | cattle - milk | β-lactam |  |
|  |  | human | human - mucous | macrolide |  |
| 2022 | Widespread of Potential Pathogen-Derived Extracellular Vesicles Carrying Antibiotic Resistance Genes in Indoor Dust | environment | dust | aminoglycoside, macrolide, β-lactam, sulfonamide, multidrug, quinolone, ansamycin, chloramphenicol |  |
|  |  |  |  |  |  |
|  |  |  |  |  |  |
|  |  |  |  |  |  |
|  |  |  |  |  |  |
|  |  |  |  |  |  |
| 2023 | Newly Detected Transmission of blaKPC-2 by Outer Membrane Vesicles in *Klebsiella pneumoniae* | human | human - isolate | β-lactam |  |
| 2023 | Planktonic and Biofilm-Derived *Pseudomonas aeruginosa* Outer Membrane Vesicles Facilitate Horizontal Gene Transfer of Plasmid DANN | environment | plankton | aminoglycoside |  |
|  |  |  | biofilm | aminoglycoside |  |
| 2023 | Pneumococcal Extracellular Vesicles Mediate Horizontal Gene Transfer via the Transformation Machinery | human | human - lungs | aminoglycoside |  |
| 2023 | Outer membrane vesicles mediating horizontal transfer of the epidemic blaOXA-232 carbapenemase gene among *Enterobacterales* | human | human - isolate | β-lactam |  |
| 2023 | Outer Membrane Vesicles Transmitting blaNDM-1 Mediate the Emergence of Carbapenem-Resistant Hypervirulent *Klebsiella pneumoniae* | human | human - blood | β-lactam, aminoglycoside |  |
|  |  |  |  |  |  |
| 2024 | Role of membrane vesicles in the transmission of vancomycin resistance in *Enterococcus faecium* | human | human - rectum | glycopeptide |  |
| 2024 | ESBL-*Escherichia coli* extracellular vesicles mediate bacterial resistance to β-lactam and mediate horizontal transfer of blaCTX-M-55 | environment | farm | β-lactam |  |
| 2024 | Membrane vesicles derived from *Enterococcus faecalis* promote the co-transfer of important antibiotic resistance genes located on both plasmids and chromosomes | animal | swine - faeces | florfenicol, oxazolidinone, macrolide, tetracycline |  |
|  |  |  |  |  |  |
|  |  |  |  |  |  |
|  |  |  |  |  |  |
| 2024 | Outer membrane vesicles secreted from *Actinobacillus pleuropneumoniae* isolate disseminating the floR resistance gene to Enterobacteriaceae | animal | swine - lungs | florfenicol |  |

Table S2: Comparison of HGT frequencies among vesicle-mediated transfer, conjugation, and transformation

| Donor of BEVs | Recipient | Vesicle-mediated HGT frequency | Transformation | Conjugation | Reference |
| --- | --- | --- | --- | --- | --- |
| *Neisseria gonorrhoeae* FA589 | *Neisseria gonorrhoeae* JS1 | 4×10^-3^-1.1×10^-2^ | / | / | [2] |
| *Neisseria gonorrhoeae* 31426 | *Neisseria gonorrhoeae* JS1 | 1.4×10^-3^-3.3×10^-3^ | / | / | [2] |
| *Escherichia coli* O157:H7 (ATCC 43895) | *Escherichia coli* JM109 | 3×10^-10^ | / | / | [3] |
| *Acinetobacter baylyi* JV26 | *Escherichia coli* DH5α | 3×10^-8^ | <10^-9^ | / | [4] |
| *Acinetobacter baylyi* JV26 | *Acinetobacter baylyi JV26* ACIAD2756::aacC1 | 1×10^-6^ | <10^-10^ | / | [4] |
| *Acinetobacter baumannii* (A_115) | *Acinetobacter baumannii* ATCC 19606 | 0.9×10^-6^-1.86×10^-5^ | 0 | / | [5] |
| *Escherichia coli* O104:H4 C227-11øcu | Enterobacteriaceae （17 species) | 7.9×10^-8^-2.8×10^-7^ | 0 | / | [6] |
| *Escherichia coli* SCAO22 | *Escherichia coli* C600 | 4.17×10^-6^ | 0 | / | [7] |
| *Klebsiella pneumoniae* (CR-HvKP) NUHL30457 | *Klebsiella pneumoniae* ATCC700603 | 1.4×10^-4^-7.58×10^-4^ | 0 | / | [8] |
| *Avibacterium paragallinarum* P4chr1 | *Avibacterium paragallinarum* Modesto | 1.74×10^-7^~5.42×10^-7^ | / | / | [9] |
| *Klebsiella pneumoniae* OXA-232-Kp | *Escherichia coli* MG1655 hph ΔhsdR | 1.12-2.31×10^-7^ | / | / | [10] |
| *Klebsiella pneumoniae* OXA-232-Kp | *Klebsiella pneumoniae* TU37-vf Δwza | 0.56-4.47×10^-7^ | / | / | [10] |
| *Klebsiella pneumoniae* (CRKP) | *Klebsiella pneumoniae* ATCC 10031 | 9.79 × 10^-9^ | / | / | [11] |
| *Klebsiella pneumoniae* (CRKP) | ESBL-producing *Klebsiella pneumoniae* ATCC 700603 | 1.42 × 10^-6^ | / | / | [11] |
| *Klebsiella pneumoniae* (CRKP) | *Klebsiella pneumoniae* hvKP NTUH-K2044 | 7.83 × 10^-6^ | / | / | [11] |
| ESBL-*Escherichia coli* SCAE22 | *Escherichia coli* EC600 | 10^-5^ | 10^-6^ | 10^-4^ | [12] |
| *Enterococcus faecalis* CQ20 | Enterococcus faecalis SC032 | 4.2 × 10^-5^ | 0 | / | [9] |
| *Porphyromonas gingivalis* 49417 | *Porphyromonas gingivalis* 33277 | 1.9 × 10^-7^ | / | / | [13] |
| *Actinobacillus pleuropneumoniae* GD2107 | *Escherichia coli* EC600 | 4.5× 10^-4^ | 0 | / | [14] |
| *Actinobacillus pleuropneumoniae* GD2107 | *Escherichia coli* BL21-pET28a | 8.2 × 10^-4^ | 0 | / | [14] |
| ESBL-*Escherichia coli* SCAE22 | *Escherichia coli* MG1655, EC600; *Proteus vulgaris* CMCC49027, *Salmonella enteritidis* ATCC14028, *Proteus mirabilis* CMCC49005, *Listeria monocytogenes* ATCC19115, *Klebsiella pneumoniae* CMCC46117, and *Escherichia coli* isolated with different ST-types (ST131, ST2582, ST48, ST115, ST156, ST162, ST2374, ST167, ST48, ST10, and ST155) . | 1.10 × 10^-7^-7.9 × 10^-6^ | / | / | [12] |
| *Klebsiella pneumoniae*-pGR | *Pseudomonas aeruginosa* ATCC 13388 | 1.6× 10^4^ CFU/μg^1^ | 0 | / | [15] |
| *Klebsiella pneumoniae*-pGR | *Burkholderia cepacia* ATCC 25416 | 1.8× 10^4^ CFU/μg | 0 | / | [15] |
| *Klebsiella pneumoniae*-pGR | *Klebsiella pneumoniae* ATCC 10031 | 2.8× 10^4^ CFU/μg | 0 | / | [15] |
| *Klebsiella pneumoniae* CRK3022 | *Klebsiella pneumoniae* K20809 | 8.2×10^4^ CFU/μg | 0 | / | [16] |
| *Klebsiella pneumoniae* CRK3022 | *Escherichia coli strain EC600* | 4.5 × 10^4^ CFU/μg | 0 | / | [16] |
| *Escherichia coli* DH5α | *Escherichia coli* BL21 | 2.76×10^6^ CFU/μg | 0 | / | [17] |
| *Pseudomonas aeruginosa* PAO9505 | *Pseudomonas aeruginosa* PAO9503 | 1.16-3.64×10^4^ /2×10^9^ vesicles ^2^ | / | / | [18] |

1: Transformation efficiency calculated as the number of transformants normalized to input BEVs DNA (CFU/μg).

2: transformation effectiveness represents the number of transformants obtained from an input of 10^9^ vesicles

# References

1. De Langhe N, Van Dorpe S, Guilbert N, Vander Cruyssen A, Roux Q, Deville S *et al.* Mapping bacterial extracellular vesicle research: Insights, best practices and knowledge gaps. *Nat Commun* 2024;**15**:9410.

2. Dorward DW, Garon CF, Judd RC. Export and intercellular transfer of DNA via membrane blebs of *Neisseria gonorrhoeae*. *J Bacteriol* 1989;**171**:2499-505.

3. Yaron S, Kolling GL, Simon L, Matthews KR. Vesicle-mediated transfer of virulence genes from *Escherichia coli* o157:H7 to other enteric bacteria. *Appl Environ Microbiol* 2000;**66**:4414-20.

4. Fulsundar S, Harms K, Flaten GE, Johnsen PJ, Chopade BA, Nielsen KM. Gene transfer potential of outer membrane vesicles of *Acinetobacter baylyi* and effects of stress on vesiculation. *Appl Environ Microbiol* 2014;**80**:3469-83.

5. Chatterjee S, Mondal A, Mitra S, Basu S. *Acinetobacter baumannii* transfers the blandm-1 gene via outer membrane vesicles. *J Antimicrob Chemother* 2017;**72**:2201-07.

6. Bielaszewska M, Daniel O, Karch H, Mellmann A. Dissemination of the blactx-m-15 gene among *Enterobacteriaceae* via outer membrane vesicles. *J Antimicrob Chemother* 2020;**75**:2442-51.

7. Li C, Wen R, Mu R, Chen X, Ma P, Gu K *et al.* Outer membrane vesicles of avian pathogenic *Escherichia coli* mediate the horizontal transmission of blactx-m-55. *Pathogens* 2022;**11**:481.

8. Li P, Luo W, Xiang TX, Jiang Y, Liu P, Wei DD *et al.* Horizontal gene transfer via omvs co-carrying virulence and antimicrobial-resistant genes is a novel way for the dissemination of carbapenem-resistant hypervirulent *Klebsiella pneumoniae*. *Front Microbiol* 2022;**13**:945972.

9. Xu J, Mei C, Zhi Y, Liang Z-x, Zhang X, Wang H-j. Comparative genomics analysis and outer membrane vesicle-mediated horizontal antibiotic-resistance gene transfer in *Avibacterium paragallinarum*. *Microbiol Spectr* 2022;**10**

10. Shen Z, Qin J, Xiang G, Chen T, Nurxat N, Gao Q *et al.* Outer membrane vesicles mediating horizontal transfer of the epidemic bla(oxa-232) carbapenemase gene among *enterobacterales*. *Emerg Microbes Infect* 2024;**13**:2290840.

11. Tang B, Yang A, Liu P, Wang Z, Jian Z, Chen X *et al.* Outer membrane vesicles transmitting bla(ndm-1) mediate the emergence of carbapenem-resistant hypervirulent *Klebsiella pneumoniae*. *Antimicrob Agents Chemother* 2023;**67**:e0144422.

12. Xu H, Tan C, Li C, Li J, Han Y, Tang Y *et al.* Esbl-*Escherichia coli* extracellular vesicles mediate bacterial resistance to β-lactam and mediate horizontal transfer of blactx-m-55. *Int J Antimicrob Agents* 2024;**63**:107145.

13. Ho M-H, Chen C-H, Goodwin JS, Wang B-Y, Xie H. Functional advantages of *Porphyromonas gingivalis* vesicles. *PLoS One* 2015;**10**:e0123448.

14. Xu M, Ke H, Zang Y, Gou H, Yang D, Shi K *et al.* Outer membrane vesicles secreted from *Actinobacillus pleuropneumoniae* isolate disseminating the flor resistance gene to *Enterobacteriaceae*. *Front Microbiol* 2024;**Volume 15 - 2024**

15. Dell'Annunziata F, Dell'Aversana C, Doti N, Donadio G, Dal Piaz F, Izzo V *et al.* Outer membrane vesicles derived from *Klebsiella pneumoniae* are a driving force for horizontal gene transfer. *Int J Mol Sci* 2021;**22**

16. Wang Z, Wen Z, Jiang M, Xia F, Wang M, Zhuge X *et al.* Dissemination of virulence and resistance genes among *Klebsiella pneumoniae* via outer membrane vesicle: An important plasmid transfer mechanism to promote the emergence of carbapenem-resistant hypervirulent *Klebsiella pneumoniae*. *Transbound Emerg Dis* 2022;**69**:e2661-e76.

17. Qiao W, Wang L, Luo Y, Miao J. Outer membrane vesicles mediated horizontal transfer of an aerobic denitrification gene between *Escherichia coli*. *Biodegradation* 2021;**32**:435-48.

18. Johnston EL, Zavan L, Bitto NJ, Petrovski S, Hill AF, Kaparakis-Liaskos M. Planktonic and biofilm-derived *Pseudomonas aeruginosa* outer membrane vesicles facilitate horizontal gene transfer of plasmid DNA. *Microbiol Spectr* 2023;**11**:e0517922.
